# Supplementary material for: Are carbon emissions trading and green financial instruments synergistic? -Comprehensive quantitative research based on content analysis
Source: PLoS One. 2024 Mar 7;19(3):e0298601. doi: 10.1371/journal.pone.0298601 (PMC10919601; doi:10.1371/journal.pone.0298601)
Supplement: S1 Data — (ZIP) [file pone.0298601.s001.zip › Data/地级市Z_F工作报告.zip!/░▓╗╒/╨√│╟╩╨/└┤╫╘╣1⁄2╓┌║┼í╛┬φ┐╦╩2╛▌═°í┐_2013.docx]

——2013年1月8日在宣城市第三届人民代表大会第四次会议上

宣城市人民政府市长	韩 军

各位代表：

现在，我代表市人民政府，向大会报告政府工作，请予审议，并请市政协委员和列席会议的其他同志提出意见。

一、攻坚克难，奋力拼搏的2012年

刚刚过去的2012年，宏观环境复杂多变，经济下行压力加大。全市人民在市委的坚强领导下，认真贯彻落实省委、省政府的决策部署，牢牢把握“稳中求进、进中求快、快中求好”的工作基调，以“招商引资突破年”、“城市建设全面推进年”、“深化作风建设年”活动为抓手，凝心聚力、攻坚克难，全力以赴稳增长、调结构、惠民生、促和谐，保持了稳中趋好的发展势头，巩固发展了和谐稳定的社会局面。

初步预测，全年实现地区生产总值760亿元，增长13%左右。财政收入137.6亿元，增长16%。固定资产投资790亿元，增长25%以上。规模工业增加值300亿元，增长17%以上。社会消费品零售总额265亿元，增长16%以上。进出口总额13亿美元，增长30%以上。城市居民人均可支配收入20600元，增长15%左右。农民人均纯收入9200元以上，增长18%左右。以上指标增幅均超过全省平均水平。全面完成省定节能减排任务。

一年来，我们突出抓了以下八个方面工作：

（一）全力支持实体经济发展。始终把发展实体经济作为稳增长的坚实基础，千方百计帮助企业排忧解难。强化政策扶持。先后出台促进工业经济平稳运行、金融支持实体经济发展、促进外贸加快发展等政策措施。设立工业发展专项资金和中小企业贷款风险补偿资金。下调困难企业四项社会保险费率。帮助524户企业争取到省困难企业政策扶持。强化重点帮扶。坚持市领导联系帮扶重点企业制度，实施“双百”帮扶行动和外贸企业“包保”服务。举办建材及酒类产品供需对接等活动。强化要素保障。积极争取用地指标，批准用地面积居全省前列。加大批而未用和闲置土地处置力度，土地例行督察整改顺利通过验收。大力推进标准化厂房建设，累计开工建设155.8万平方米，竣工60万平方米。组织30多场银企对接活动，新增贷款站上新的台阶，全年达到110.7亿元。争取全国社保基金9亿元贷款，市开发区基础设施建设利用世行贷款示范项目通过预评审，均为全省唯一。市国投公司公司债、中鼎集团中期票据及宣酒集团中小企业私募债券成功发行。凤形耐磨、五星食品、聚隆机械、广信农化4家企业上市报会待审，宣纸集团等企业上市工作加快推进。举办58场招聘会，组织250余户企业与皖北地区进行就业对接，缓解了企业用工困难。强化运行调节。改进工业发展考核办法，建立重点项目建设调度和考核制度。加强经济运行监测，实施即时调度、旬调度、月调度和联合调度，促进经济即期增长。

（二）积极推进产业转型升级。始终把转型升级作为稳中求进的重要内容，加快转变经济发展方式。工业转型取得新成效。战略性新兴产业产值增长30%以上，高新技术产业增加值增长27%左右。新建投产规模企业79户。企业自主创新能力进一步增强，新增省级工程技术研究中心3家、企业技术中心7家，开发省级高新技术产品149项。宁国橡塑密封件产业基地被认定为国家火炬计划特色产业基地。博瑞特公司建成全国同行业首家院士工作站。宁国耐磨材料、泾县电机、绩溪不锈钢等3个省级检验检测中心投入使用。完成工业技改投资150亿元，115个投资过千万元的技改项目完工。品牌战略深入实施，新增中国驰名商标3件、省著名商标13件、省名牌产品15个。服务业发展实现新突破。服务业增加值244亿元，增长11%左右。成功获批国家农产品现代流通综合试点城市，宣州成为省级服务业综合改革试点区。双桥物流园区建设初见成效，百汇商贸物流园一期建成开业，农副产品批发市场一期基本建成，纺织服装批发市场等项目开工建设。麦莎广场、八佰伴百货正式开业。宣城文教创意产业园建设有序推进，文房四宝交易中心主体工程即将竣工。宣城日报文化传媒有限公司组建完成。恩龙世界木屋村整体提升工程和中国宣纸传习基地、绩溪文化体育创意产业园等项目加快建设。旅游业持续快速发展，游客接待量、旅游总收入分别增长43.3%和54.1%。龙川成功创建国家5A级旅游景区。广德木子度假村、宁国伯爵王朝、绩溪锦江上河国际等一批高星级酒店投入运营。新港生态湖旅游综合开发等项目进展顺利。新增省级休闲农业与乡村旅游示范县1个、最佳旅游乡镇3个、优秀旅游乡镇3个、五星级农家乐5家。金融业稳步发展，新开业金融机构和网点11家，小贷公司增资扩股4.9亿元。市邮政管理局挂牌成立。节能减排获得新进步。万元工业增加值能耗下降8%以上。12个资源节约和环保项目列入中央投资计划，47个节能节水和资源综合利用项目列入省投资导向计划。组织实施145个减排项目，环保“绿剑行动”深入开展，污染排放量稳步下降。

（三）加大承接产业转移力度。始终把承接产业转移作为促进转型发展的重要助推器，努力打造新的增长极。“招商引资突破年”活动成效明显。大力推广郎溪县招商引资经验，进一步放大“郎溪现象”。积极拓展招商领域。深化与苏浙沪合作园区建设，加强与央企、知名民企合作，积极参与皖粤经贸合作等活动。新签亿元以上省外投资项目520个，到位资金485亿元。实际利用外资4.2亿美元，增长33%。市本级成立6个产业招商组，签约项目47个，协议引资216亿元。园区扩容升级步伐加快。宣城和宁国开发区积极争创国家级经开区和省级高新区。宁国、宣州等4个开发区扩区工作稳步推进。郎溪开发区通过省级高新区专家组评审，经都产业园被授予“中国纺织产业转移试点园区”称号。广德开发区荣获省级新型工业化产业示范基地、循环经济园区和“两化”融合示范区称号。宁国港口生态工业园成为省新型工业化产业示范基地。重点项目建设加速推进。在建亿元以上项目489个，累计完成投资430亿元。“861”项目开工、竣工数和完成投资量占年度计划比例均居全省前列。市开发区裕丰数控机床等项目开工建设，中鼎产业园、星亚电动车等项目进展加快。国投宣城电厂二期、南漪湖风电场、北京中科通用（宣城）环保电厂等项目启动建设。上海通用汽车（广德）研发试验中心建成运营。宁国司尔特缓释复合肥搬迁扩建项目试生产。绩溪抽水蓄能电站正在筹备开工。

（四）扎实开展美好乡村建设。始终把“三农”工作摆在重要位置，在全省率先启动和顺利推进美好乡村建设，认真组织实施“6611”行动计划，完成87个美好乡村创建任务，综合整治340个自然村，提升10条示范带。现代农业加快发展。粮食总产创历史新高。市粮食储备库一期投入使用。农业产业化“262”工程深入实施，实现农产品加工产值420亿元，增长20%。3个省级农业产业化示范区产值均突破50亿元，处于全省前列。新增部、省级畜禽养殖标准化示范场区10个。新增农民专业合作社示范社国家级3个、省级14个，居全省首位。新增“三品”认证农产品12个。成为首批“中国森林食品产业示范基地”。启动“森林增长工程”和国家森林城市创建工作，新增造林15.2万亩。农民生活条件不断改善。进一步完善县域城镇体系规划，修编村庄布点规划。改造农村危房1.7万余户。完成45座农村危桥加固改造，升级改造县乡道100公里。建设农村饮水安全工程99处，解决了16万农村居民饮水安全问题。农村清洁工程强力推进。落实保洁队伍、环卫设施、经费保障和长效机制，新增垃圾箱7.9万只、垃圾池1.3万座、焚烧炉116个、中转站65个、垃圾清运车900辆和污水处理设施92处，聘用保洁人员7500多人，基本实现农村垃圾处理全覆盖。生态市创建成效显著，创建国家环保模范城市规划通过环保部评审，绩溪、宁国创成国家级生态县（市），郎溪、广德、泾县和旌德成为首批省级生态县。

（五）大力完善城乡基础设施。始终把基础设施建设作为增强承载力的重要工作，促进发展环境不断优化。“城市建设全面推进年”活动扎实开展。启动新一轮城市总体规划修编，编制完善城市停车场等12个专项规划。加快中心城市建设，市本级完成投资130亿元。彩金湖新区建设正式启动。新建续建 41条道路，15条道路建成通车。北门旧城改造等项目加快推进。市体育馆、图书馆、规划馆动工建设。海关、出入境检验检疫机构项目完成征迁。国家园林城市创建工作有力启动，宛陵湖西湖景区主体工程和敬亭湖公园基本完工，泥河、道叉河正在整治；完成主城区29条道路绿化提升改造，新增绿地面积120多万平方米。城管执法工作进一步加强。交通建设有力推进。完成建设投资40亿元，增长37.7%。芜雁高速建成通车，宣广高速宣城东、西出入口改扩建完工并使用。宣宁高速路面工程完成过半，宁绩高速路基工程基本完成，宁千高速路基全面施工。宣南铜高速复工建设，溧广高速基本完成开工准备。宣狸一级公路完成路基和部分路面工程。梅岭隧道、省道217绩长段改造主体工程进入收尾。芜申运河宣州段基本完成，郎溪段开工建设。宣狸、广宁高速和省道322宣泾段、省道214定十段一级公路改造以及水阳江航运综合开发等工程前期工作积极推进。合福高铁建设进展顺利，皖赣铁路扩能改造和杭黄、商合杭铁路以及芜宣机场筹建前期工作扎实开展。水利、电力等设施继续改善。完成水利建设投资17亿元，增长23.2%。水阳江下游近期防洪治理工程全面开工。宋墩排涝泵站和溪口枢纽除险加固工程基本完工。双桥联圩、敬亭圩部分堤防和郎溪中斗闸除险加固工程加快推进。7个中小河流治理项目、179项重点农田水利建设工程和一批水库除险加固工程顺利完成。县市区全部荣获省“江淮杯”表彰，郎溪成为“全国农田基本建设先进县”。抗击台风“海葵”和防汛抢险取得全面胜利，受到省委、省政府表彰。220千伏桃州变扩建和3个110千伏输变电工程建成投产，2个220千伏和宣城中心变等4个110千伏输变电工程加快建设。农网升级改造和通信基础设施建设积极推进。

（六）着力保障和改善民生。始终把保障和改善民生作为政府工作的最大责任，努力解决群众最关心、最直接、最现实的突出问题。省定33项民生工程全面完成。财政民生支出144.4亿元，增长26%，占财政支出的80.9%，其中用于民生工程建设投入24.8亿元。保障性住房建设工作位居全省前列，市本级开工保障性住房和安置房160万平方米。住房公积金归集面进一步扩大，安全有效管理得到加强。新建、改扩建农村敬老院50所，农村五保集中供养率接近60%。市区新改扩建大棚蔬菜基地1000亩。就业和社会保障工作不断加强。新增就业6.1万人，下岗失业人员再就业1.5万人，城镇登记失业率3.55%，基层就业和社会保障平台建设基本实现全覆盖。圆满完成五项社会保险扩面征缴任务，城乡居民养老保险工作受到国务院表彰。城乡低保标准进一步提高。全面实施高龄老人津贴发放制度。加强扶贫开发工作，累计脱贫1.2万人。市本级和4个县社会（儿童）福利中心主体工程完工。马山生态陵园开工建设。文教卫体等社会事业加快发展。我市再次被评为“全国文化体制改革工作先进地区”。全面完成乡镇综合文化站建设任务。实现村村有书屋、村村通广播电视。扎实开展农村义务教育学校标准化建设。中小学校舍安全工程和农村留守儿童之家建设全部完成。加强学前教育工作，学前三年毛入园率处于全省前列。宣城中学新校区主体工程完工。2所职业教育学校被列入国家中职教育改革发展示范项目。修订颁布全民健身实施计划，在省第三届体育大会上取得良好成绩。继续完善基层医药卫生体制改革，启动县级公立医院综合改革，药品零差率销售全面实施。巩固发展新型农村合作医疗。人口计生工作进一步加强，低生育水平、出生人口性别比等指标继续保持全省领先。制定出台政策措施，着力引进高层次、高技能人才。市残疾人康复中心、防震减灾中心和地震台网建设稳步推进。民族团结工作不断加强，宗教事务依法管理进一步规范。圆满完成社区居委会统一换届选举工作。颁布实施妇女儿童新一轮十年发展规划。残疾人、老龄和红十字、慈善福利事业进一步发展。统计、物价、外事侨务、新闻出版、档案、地方志、机关事务、气象等工作取得新进展，税务、烟草等工作迈出新步伐。

（七）切实加强和创新社会管理。始终把加强和创新社会管理摆在突出位置，努力使社会既充满活力又和谐稳定。开展重大事项社会稳定风险评估，健全排查预警等制度，社会管理创新“十大体系”建设持续推进。启动实名制信息共享与服务平台建设，实施居住证制度。法律援助受援率位居全省之首。在全省率先实现社区矫正机构全覆盖。平安宣城建设继续深化，立体化治安防控体系建设深入推进，严厉打击各类违法犯罪活动。扎实推进领导干部开门接访、带案下访和“六个一”大走访活动，妥善化解了一批信访积案。市医疗纠纷调处中心挂牌成立。市区一体化群众来访联合接待服务中心建成使用。所有煤矿关闭到位，烟花爆竹生产企业有序退出，校车安全、食品药品安全等监管力度持续加大。深化“三个机制”衔接试点工作，强化应急队伍集训演练，推进人防（应急）指挥中心建设。坚持军民融合发展，国防教育、国防动员工作进一步加强。在全省率先出台征兵工作实施办法，圆满完成年度征兵任务。绩溪荣获“全国双拥模范县”称号。

（八）努力提高政府工作水平。始终把政府自身建设作为推进各项工作的重要保障，努力做到“提神、提速、提效”。扎实开展“深化作风建设年”活动，实行基本建设项目并联审批、重大投资项目审批“绿色通道”制度，强化重点引资项目评审调度，建立帮办服务体系。深化民主考评活动，启动新一轮投资环境集中整治工作。坚持依法行政，进一步规范行政决策。积极推进政务公开、政务服务标准化。自觉接受市人大和市政协监督，累计办理市人代会议案、人大代表建议和政协委员提案281件。加强与各民主党派、工商联和无党派人士、人民团体的联系。完善廉政风险防控体系。加强工程建设领域突出问题专项治理。深入实施“阳光村务”工程。加快公共资源交易平台建设。深化经济责任审计和重点项目、重点资金跟踪审计，社保资金项目审计工作获得国家审计署、人社部表彰。

各位代表，过去的一年，面对复杂严峻的经济形势，面对自然灾害的不利影响，我们迎难而上、砥砺奋进，取得了来之不易的成绩。尤其是合工大宣城校区一期工程顺利建成、按期开学，创造了为人称道的“宣城速度”，彰显出“创新、拼搏、合作、奉献”的新时期“宣城精神”。这是省委、省政府和市委坚强领导的结果，是市人大、市政协监督支持的结果，是全市人民团结奋斗、共克时艰的结果。在此，我谨代表市人民政府，向全体人大代表、政协委员，向全市广大工人、农民、知识分子、干部，向驻宣部队、武警和消防官兵、政法干警，向各民主党派、工商联、人民团体，向关心、支持宣城发展的各界人士，表示衷心的感谢和崇高的敬意！

在看到成绩的同时，我们也清醒认识到，经济社会发展还存在不少困难和问题。主要是：持续加快发展的基础还不稳固，要素制约较为明显，经济下行压力加大，部分指标和项目未能实现预定计划；全社会产能过剩，但我市经济总量还偏小，产业不大、结构不优问题相对突出；县域经济发展不平衡，市本级辐射和带动力不强；居民收入和社会保障水平还不高，保障和改善民生的任务繁重；行政审批过多、服务效率不高的现象比较明显。对此，我们必须凝聚力量，下定决心，广拓资源，采取更加有力的措施，切实加以解决。

二、锐意进取，扎实做好2013年各项工作

2013年是深入贯彻落实党的十八大精神的开局之年，是为全面建成小康社会奠定坚实基础的重要一年。前进的征程中有很多机遇，一路伴行的肯定也有不少挑战。我们要深入贯彻落实党的十八大和中央、全省经济工作会议精神，继续坚持“稳中求进、进中求快、快中求好”的基调，按照“全部工作以经济建设为主、经济建设以工业突破为主、工业突破以招商引资为主、招商引资以项目进园区为主”的方针，以“招商引资年”、“工业突破年”、“项目推进年”、“城市建设年”、“交通会战年”为抓手，加快推进转型发展、创新发展、统筹发展、开放发展、和谐发展。

全市经济社会发展的主要预期目标建议为：地区生产总值增长13%；规模工业增加值增长17%以上；服务业增加值增长12%；财政收入增长16%以上；固定资产投资增长25%；社会消费品零售总额增长16.5%；进出口总额增长20%；城市居民人均可支配收入增长15%；农民人均纯收入增长16%。确保完成省定节能减排任务。实现上述目标，工作中必须重点抓好以下十个方面：

（一）扩大有效投入，进一步增强发展后劲。坚持以产业、项目为支撑，扩大有效投入，是现阶段宣城加速发展的重中之重。聚力推进招商引资。始终把招商引资作为经济工作的第一要事，进一步改进招商方式，壮大招商队伍，完善工作机制，严格考核奖惩，优化招商环境，推进招商引资实现更大突破。坚持全员招商和专业招商并重、外资和内资并重、先进制造业招商和现代服务业招商并重，加强产业招商、集群招商、驻点招商、以商引商，强化跟踪跟进，增强主动性和实效性。建立健全区域合作交流机制，巩固完善长三角招商网络，进一步加大珠三角招商力度。积极拓展境外招商，在引进港澳台资本上寻求新突破，在日本、韩国、新加坡等区域招商取得新进展。注重择商选资，紧盯世界500强、国内100强、行业前10强等大企业、大项目。确保亿元以上项目实际到位资金、实际利用外资分别增长25%和20%。狠抓项目落地、开工和建设，提高资金到位率、项目开工率和投产达效率。着力拓宽融资渠道。强化银政企对接，力争新增贷款达到120亿元。积极争取世行、亚行、国际金融公司和外国政府贷款。实施市开发区利用世行贷款示范项目。进一步扩大直接融资。推进在审企业上市工作，加快宣纸集团、黄山胶囊、华卫禽业等企业上市步伐。做大市国投公司，做实市交通投资公司，组建市工业投资公司，增强政府融资平台融资能力。积极争发中小企业集合债券、企业中期票据，抓好市国投公司二期公司债券、市开发区建投公司企业债券等发行工作。积极引进股权投资基金和创业（风险）投资基金。加大信托、租赁融资力度。全力谋划争取项目。深入研究国家产业政策和投资导向，在主导产业、重大基础设施、民生工程、节能减排和生态建设等领域，认真谋划一批大项目、好项目，力争更多项目进入国家和省投资计划盘子。

（二）突出工业强市，进一步加快新型工业化步伐。深入实施工业“5+3”工程和“千亿产业”计划，促进工业结构优化升级。做大做强现有企业。继续实施中小企业成长工程，完善中小企业服务体系，重点扶持50户“专精特新”企业跨入亿元行列、100户以上成长性企业进入规模企业，力争新建投产规模企业80户。大力扶持本土企业发展，鼓励企业兼并重组，壮大实力。严格落实惠企减负政策，坚持市领导联系帮扶、“双百”帮扶等制度，强化运行监测和要素保障，健全工业项目推进机制，积极帮助企业开拓市场。改造提升传统产业。编制首位产业发展规划。全面实施产业改造升级计划，组织实施200个重点工业项目、200个重点技改项目。确保博迪新材料、宣州精方药业改造、郎溪博朗凯德车用面料、广德涌诚机械二期、宁国保隆汽车气门嘴生产线、泾县和森钢管、绩溪泰昂电力二期等项目投产。加快国投宣城电厂二期、中鼎动力、宣酒小窖酿造基地、广德PCB产业园、宁国源光电器改造、泾县宝泰金属复合材料、旌德黄山胶囊扩建等项目建设。力争宣城海螺水泥二期、金达利液压元件二期等项目开工。加快发展新兴产业。加强政策扶持和绩效考核，培育发展战略性新兴产业，力争产值增长25%。加快推进绩溪抽水蓄能电站、南漪湖风电场、北京中科通用（宣城）环保电厂等项目建设。大力实施振兴皖药行动计划，推进新兴药械产业园建设。促进开发区转型发展。优化开发区管理机制，实施开发区发展成效考核，完善发展条件和环境，引导关联产业集聚发展，培育发展主导产业。加快4个省级开发区扩区和9个筹建省级开发区“转正”步伐。大力推进市开发区建设发展，建成投产一批产业层次高、带动力强的大项目、好项目，新增一批规模工业企业，力争升格为国家级开发区。支持宁国和广德、郎溪开发区争创国家级开发区。继续推进标准化厂房建设，吸引更多企业入园创业。

（三）坚持城乡统筹，进一步加速新型城镇化进程。全面推进新型城镇化“11134”工程，加快构建科学合理的现代城镇体系，促进城乡统筹发展。大力推进中心城市建设。市本级完成投资150亿元以上。科学修编城市总体规划。继续推进主城区“一城四片区”和彩金湖新区建设。续建和新改建36条城市道路，完善城区“二环八射”路网，进一步优化城区交通。加快合工大宣城校区二期、市体育馆、北门旧城改造等项目建设。扎实推进国家园林城市创建工作。完成市县城市绿道规划编制，实施皖南“金项链”区域景观绿道工程。完成宛陵湖西湖景区建设。继续做好宛溪河综合整治二期工程前期工作。加快城市内河整治步伐。开展“数字城管”二期建设，提高城市管理水平。加快建设县城和中心镇。坚持错位发展，彰显地域特色，积极培育县级中等城市。以省级扩权强镇试点镇和省级中心镇为重点，加快建设一批特色镇和经济强镇。强化城镇基础设施和公共服务设施建设，增强综合承载力，提升城镇品位。加快户籍制度改革，有序推进农村转移人口市民化。扎实开展“交通建设会战年”活动。完成交通基础设施建设投资60亿元以上。确保完成宣宁高速和宣狸一级公路、省道217绩长段、梅岭隧道和国道205榔树口至雀岭示范工程，加快宁绩、宁千、溧广、宣南铜高速和芜申运河定埠段建设进度。开工建设省道322宣泾段、省道214定十段和国道205泾南段、省道323滑渡至新桥、省道215绩溪至歙县段一级公路项目。力争皖赣铁路扩能改造工程（芜宣段）及宣城火车站升级改造、宣狸和广宁高速、水阳江航运综合开发及宣城、郎溪、宁国客运总站等项目开工。继续保障合福高铁建设。推进芜黄高速和商合杭等铁路、芜宣机场建设前期工作。积极推进城乡客运一体化。加强水利、电力等设施建设。完成水阳江下游近期防洪治理主体工程和溪口闸改建工程。加快中斗闸、双桥联圩和敬亭联圩堤防除险加固及渠系整治。开工建设宛溪河中小河流治理、关庙水库和双塘泵站项目。力争开工青弋江综合治理项目。积极推进汤村等6座中型水库前期工作，加快实施青弋江灌区续建配套与节水改造。继续改善电力基础设施，确保220千伏梅丰、太平和110千伏宣城中心变等5项输变电工程竣工投产，开工建设220千伏梓山变等输变电工程。开工建设多普勒气象雷达项目。

（四）建设美好乡村，进一步开创“三农”工作新局面。将美好乡村建设作为“三农”工作的有力抓手，促进农业增效和农民增收，建设幸福美好家园。增强农业综合生产能力。继续改善农业生产条件，实现粮食稳定增产。启动市粮食储备库二期和皖东南粮食批发市场建设。继续实施畜牧业升级计划，推进畜禽养殖标准化示范区创建。加速低产茶园改造和标准高效茶园建设，提升茶产业质量和水平。加快培育油茶、苗木花卉、山核桃、竹业等林特产品生产基地。扩大优质烟叶种植规模。继续加强“菜篮子”工程建设，新建市区大棚蔬菜基地1000亩。提升农业产业化经营水平。培育和扶持龙头企业、农民专业合作社、专业农户等农村新型经营主体。积极稳妥推进农村土地承包经营权流转。加快现代农业示范区和农业产业化示范区建设，力争新增3个省级现代农业示范区，支持宣州创建国家级现代农业示范区。推进农产品精深加工，确保立大集团溜味族食品基地建成投产，加快山里仁食品工业园等项目建设，开工建设绩溪丰润公司生猪屠宰加工、刘郎食品熟食生产等项目。重点帮扶200个村发展现代农业产业，打造“一村一品”特色专业村。加快休闲农业和乡村旅游发展，争创一批优秀乡村旅游示范点。创新农业服务体制机制，完善农技服务和农产品质量安全监管体系，完成77个乡镇农技站新改扩建任务，继续开展农技人员包村联户活动。全面推进美好乡村创建。深入实施规划引领、新村创建、污水治理、清洁提升等“九大行动”，巩固提升创建成果。高起点编制美好乡村建设规划，完成村庄布点规划修编，加强中心村村庄规划工作。重点培育150个中心村，整治600个自然村，提升100个特色村。深入推进10条美好乡村示范带建设，在国省道以及公路沿线重点建设100个高标准美好乡村示范点。大力整合涉农项目资金，加大美好乡村建设投入。强化农村环境卫生综合整治，积极推进国家农村环境连片综合整治项目。突出垃圾分类处理，健全保洁长效机制，推动农村垃圾处理常态运作。加强中心村污水处理工作。推进新一轮农村电网升级改造。完成200公里县乡道升级改造，加固改造40座农村危桥。改造农村危房1.77万户。加强农田水利建设，改善灌溉条件。加强安全饮水工程建设。继续推进农民文化广场建设项目。加快广播电视由“村村通”向“户户通”迈进。加快农村通信事业发展，力争有线宽带和3G入户率提升20%。

（五）优化经济结构，进一步壮大现代服务业。坚持把加快服务业发展作为优化产业结构、促进转型升级的战略重点，推动服务业特别是现代服务业发展壮大。加快提升旅游产业。积极推进皖南国际旅游文化示范区建设，组织实施市本级“6521”旅游项目工程，重点抓好敬亭山风景区文化品质提升、新港生态湖旅游综合开发和金梅岭、鳄鱼湖景区改造提升以及高星级酒店建设等项目。积极推动泾县大云岭红色景区5A创建工作，加快宣州昆山湖休闲旅游、广德卢湖竹海休闲度假区、旌德黄山东线生态旅游产业园等项目建设，支持绩溪百里历史文化生态走廊项目有序推进。加强旅游精品线路、景区营销推介，大力开发特色旅游商品。举办第六届中国（宣城）文房四宝文化旅游节。积极发展物流业和金融业。加快推进双桥物流园区建设，扩充完善道路、水电管网等配套设施，积极申报省级现代服务业集聚区。确保农副产品批发市场一期投入运营，加快建设中国茶府、纺织服装批发市场等项目，开工建设百汇二期、农副产品批发市场二期、亚夏汽车文化财富广场、冷链物流市场、联邦物流园、旅游商品博览城等项目。完善地方金融体系，完成农商银行机构改革任务，积极推进浦发银行等来宣设立机构。加快组建村镇银行，规范发展小贷公司和融资性担保公司。优化金融生态环境，加强金融生态县创建。不断繁荣城乡消费市场。加快建设城乡现代流通体系，培育壮大商贸流通企业，力争新增限额以上单位46家。扎实开展“农产品现代流通综合试点”工作。完善“五位一体”放心体系，完成“放心肉”体系建设试点市任务。大力推进“新网工程”建设。抓好星隆国际广场、大润发超市等项目建设。加快城乡农贸市场标准化建设，改造提升一批市区菜市场。建成一批农村超市。积极引导发展电子商务等新型业态。

（六）深化改革创新，进一步激发发展活力。不断加大改革力度，坚持创新驱动发展。着力增强自主创新能力。落实高新技术企业激励创新政策，培育和发展一批创新型企业。力争新增30家高新技术企业、3家省级工程技术研究中心、5家省级企业技术中心，高新技术产业增加值达到100亿元。加快创新载体建设，力争市开发区和郎溪开发区成为省级高新区。支持宁国自主创新综合试验区建设。加快市本级科技企业孵化器和“科技路路通”创新服务站建设。开工建设合工大科技创业园和国家宣纸及文化用品质量监督检验中心。深化重点领域改革。积极争取全国农村土地使用制度综合改革试点，支持宣州省级服务业综合改革试点、郎溪城乡一体化综合配套改革和广德“省直管县”试点工作。推进事业单位分类改革，开展省级经济发达镇行政管理体制改革试点。深化“营改增”等财税体制改革，深入推进行政事业单位资产统管。巩固完善医药卫生体制改革，稳步推进县级公立医院综合改革。推行城乡一体化住户调查改革。发展壮大民营经济。进一步创新非公经济发展的体制机制，认真落实关于促进民营经济发展的政策措施，放宽创业条件，强化信贷和技术扶持，加强企业家队伍建设，掀起全民创业高潮，推动民营经济大发展。提升开放型经济水平。坚持出口和进口并重，优化出口产品和市场结构，加快建设特色产品出口基地。鼓励企业通过并购、参股、境外上市等形式参与国际经济技术合作。基本完成海关、出入境检验检疫局项目主体工程，营造大通关环境。

（七）事业产业并重，进一步打造文化强市。认真落实《文化强省建设实施纲要》的各项要求，推动文化事业全面繁荣、文化产业快速发展。提升全民文明素质。大力弘扬和践行新时期“宣城精神”。加强公民道德建设，继续组织道德模范、“感动宣城”十佳人物评选等活动。深入开展省级文明城市创建工作，积极创建文明行业和文明单位。积极推进文化惠民。加快建设市图书馆、规划馆、档案馆等公共文化设施。加强文物保护利用，推进谢朓楼重建、广教寺双塔抢救维修等工程建设。积极申报省第四批非物质文化遗产项目。继续开展“送文化下乡”等活动。组织参加省第十届艺术节。加快发展文化产业。实施重大文化项目带动战略，加快推进宣城文教创意产业园和中国宣纸传习基地、绩溪文化体育创意产业园等建设。实施“文房四宝”特色文化品牌提升行动计划。大力培育文化市场主体，扶持文化企业做大做强。启动宣城日报报业集团、宣城广电传媒集团组建工作。壮大市文房四宝发展有限公司。继续推进市广电文化产业中心建设。组织实施“文化名家”、“六个一批”拔尖人才、青年文化英才扶持和基层文化人才建设工程，培养造就一批文化人才。

（八）强化环境保护，进一步推进生态文明建设。坚持以生态文明协调经济社会发展，不断提升生态竞争力，加快建设宜居宜业宜游的生态强市。加强生态保护和建设。深入推进国家生态市、环保模范城市和森林城市创建工作，支持郎溪、广德、泾县、旌德创建国家级生态县。认真实施“森林增长工程”，新增造林13万亩。强化环敬亭山区域生态保护和夏渡森林公园的保护与建设。严格水资源管理，强化城镇集中式饮用水水源地保护。加强以农村沼气为重点的清洁能源建设。大力推进节能减排。认真落实能源消费强度和总量“双控制”要求，坚决淘汰落后产能。加快66个重点节能、节水项目建设。严格执行产业投资发展导向和限制目录，严格建设项目环境准入和规划环评。大力推进城市污水管网建设，加快省级开发区、工业园区和重点乡镇污水处理厂及配套管网建设。强化脱硫脱硝设施建设和运行管理。加大机动车尾气和农业源减排力度。深入开展环保“绿剑行动”，重点加强涉重金属、化工、印染、造纸等行业和园区、环境敏感区的环境监管。强化资源节约集约利用。落实最严格的耕地保护制度，加大批而未用、闲置土地清理处置和农村建设用地整治的力度，争创国土资源执法和节约集约模范市。大力发展循环经济，加快循环经济园区和项目建设。推广绿色建筑，积极创建国家绿色生态城区和可再生能源建筑应用示范城市。倡导低碳消费理念，推广绿色消费模式。

（九）繁荣社会事业，进一步优化公共服务。扎实推进基本公共服务均等化，促进经济社会协调发展。努力办好人民满意的教育。完成学前教育三年行动计划。深入推进农村义务教育学校标准化建设和城乡学校布局调整，加强学校师资力量建设与交流，促进义务教育均衡发展。提升发展高中阶段教育。确保宣城中学新校区9月份建成使用。加强职教院校建设，增强职教院校服务经济发展的能力。推进市特教中心和体校发展。积极开展全民健身活动，举办市首届全民健身运动会。提高医疗卫生服务和保健水平。强化医疗市场监管，努力构建和谐医患关系。加强疾病预防控制和妇幼保健工作。加快宣城中心医院项目建设，开工建设市人民医院外科大楼和市精神病院项目。创新人口计生服务管理。进一步完善农村人口计生工作机制，着力推进城市和流动人口计生工作网格化管理，全面实施免费孕前优生健康检查项目。确保人口出生率控制在10‰以内，人口出生政策符合率稳定在92%以上，出生人口性别比保持正常。促进其他各项事业全面进步。加快市残疾人康复中心、防震减灾中心和地震台网建设。继续重视老龄和红十字、慈善福利事业，保障妇女和未成年人合法权益。认真做好物价、民族宗教、外事侨务、对台、新闻出版、地方志等工作。完成“十二五”规划中期评估和调整工作。认真组织开展第三次经济普查。

（十）保障改善民生，进一步增进人民福祉。坚持富民优先，继续加强民生建设，努力提升群众幸福指数。创业带动就业夯实民本。继续实施收入倍增规划，确保城乡居民收入增幅与经济发展同步。推进基层创业就业服务平台建设，重点抓好高校毕业生、下岗失业人员、返乡农民工和退役士兵等群体的就业工作。引导农村劳动力就近就地创业就业。力争新增就业3.9万人，城镇登记失业率控制在4.2%以内。健全保障体系维护民利。确保完成省定33项民生工程目标任务。扎实做好保障性住房的建设、质量和分配管理工作，加快安置房建设。市本级新开工建设各类保障性住房80万平方米以上。强化社会保险扩面征缴。探索建立失地农民生活保障长效机制。加快市本级和县市区社会保障服务中心建设。加强城乡低保工作，进一步提高保障标准和补差水平。继续实施农村敬老院改扩建工程，力争农村五保集中供养率达到90%。确保市社会（儿童）福利中心建成使用。争取马山生态陵园主体工程完工。扎实开展扶贫开发工作。创新社会管理保障民安。推进社会管理创新“十大体系”建设，完善源头治理、动态管理、应急处置相结合的工作机制。加强社区建设与管理。创新流动人口和特殊人群管理服务。加强法制宣传教育，开展全国法治城市争创活动，进一步做好法律援助和社区矫正、安置帮教等工作。坚持用群众工作统揽信访工作，深入开展领导干部接访、下访活动，加强社会矛盾纠纷排查化解，完善人民调解、行政调解、司法调解联动机制。全面实施重大事项社会稳定风险评估制度。严格落实安全生产责任制，加大隐患排查和整改力度，有效遏制重大事故发生。继续加强校车安全管理和校园周边环境整治。强化食品药品安全监管。健全突发公共事件预警和应急处置机制，增强防灾减灾和应对突发事件的能力。深化平安宣城建设，完善立体化社会治安防控体系，依法防范和惩治违法犯罪活动，保障人民生命财产安全。支持工会、共青团、妇联等人民团体参与社会管理和公共服务。发展壮大志愿者队伍。强化全民国防意识，大力支持驻宣部队建设，提升双拥优抚安置工作水平，积极争创省级双拥模范城“三连冠”，巩固和发展军政军民团结。

三、努力建设学习型、创新型、服务型政府

面对转型跨越的新形势、新任务，面对全市人民的新期待、新要求，我们将恪守“为民、务实、高效、清廉”的理念，严格执行中央八项规定，切实改进作风，密切联系群众，牢记使命、勇担责任、务实勤俭、积极作为，奋力开创政府工作新局面。

坚持开拓创新。加强学习、积极借鉴、勇于实践，不断提高政治素养和施政水平。进一步解放思想、更新观念，以争先、领先、率先的气魄，创业、创新、创优的精神，开放、开明、开拓的理念，抢抓发展机遇，用足用活政策，千方百计破解发展难题。坚持勇于担责、敢闯敢试，切实增强工作的主动性和创造力，不断提高推进科学发展能力。

坚持务实高效。进一步简政放权，优化政务服务，大幅精简行政审批事项至200项以内。实施行政审批提速工程，认真落实项目并联审批、“绿色通道”办理、限时办结、帮办服务等制度，深化民主考评工作，加强政务服务标准化、规范化建设，切实优化发展环境。强化工作落实，健全督查通报、绩效考评和责任追究机制，确保重大决策、重要部署、重点项目和目标任务的全面落实和完成。深化作风建设，多下基层调研、多进企业帮扶、多帮群众解难。精简会议和文件，讲短话、开短会、行短文，切实提升行政效能。

坚持依法行政。健全决策机制和程序，建立决策问责纠错制度。坚持问政于民、问计于民、问需于民，加强“网络政民互动”等工作，使政府决策更加科学、运作更加规范。自觉接受市人大的法律监督、工作监督和市政协的民主监督，认真办理人大议案、代表建议和政协委员提案，主动听取民主党派、工商联、无党派人士和各人民团体的意见、建议，高度重视司法监督、舆论监督和社会公众监督。深入推进依法行政示范单位创建活动。深化政府信息公开和部门办事公开，使各项制度更加健全、政务工作更加透明。

坚持清正廉明。扎实开展以为民、务实、清廉为主要内容的群众路线教育实践活动，推进惩治和预防腐败体系建设，强化廉政风险防控，自觉遵守廉洁从政各项规定，切实做到干部清正、政府清廉、政治清明。进一步加快公共资源交易平台建设，完善招投标监管制度。加强对重点工程、重点领域和经济责任的审计监督、行政监察与全程监管。深入推进节约型机关建设，严格控制“三公”经费支出，切实把有限的财力投到民生改善上。加大专项整治力度，坚决纠正损害群众利益的行为，严肃查处各种违法违纪案件。

各位代表，我们的任务艰巨而繁重，责任重大而光荣。一元复始，抒写美好希望；万象更新，承载幸福吉祥。让我们高举中国特色社会主义伟大旗帜，深入贯彻落实科学发展观，在市委的坚强领导下，紧紧依靠和团结全市人民，脚踏实地，锐意进取，投身中国梦，建设新宣城，奋力谱写经济社会发展的新篇章，为全面建成小康社会而努力奋斗！
